# Nanomaterial‐Modified Conducting Paper: Fabrication, Properties, and Emerging Biomedical Applications

**DOI:** 10.1002/gch2.201900041

**Published:** 2019-09-09

**Authors:** Saurabh Kumar, Chandra Mouli Pandey, Amir Hatamie, Abdolreza Simchi, Magnus Willander, Bansi D. Malhotra

**Affiliations:** ^1^ Centre for Nano Science and Engineering (CeNSE) Indian Institute of Science Bengaluru 560012 India; ^2^ Department of Biotechnology Delhi Technological University Main Bawana Road Delhi 110042 India; ^3^ Department of Applied Chemistry Delhi Technological University Main Bawana Road Delhi 110042 India; ^4^ Department of Science & Technology Campus Norrkoping Linkoping University SE 60174 Norrkoping Sweden; ^5^ Nanostructured & Advanced Materials Lab Department of Materials Science and Engineering Sharif University of Technology Tehran 1458889694 Iran

**Keywords:** conducting paper, deposition methods, nanomaterials, point‐of‐care devices

## Abstract

The emerging demand for wearable, lightweight portable devices has led to the development of new materials for flexible electronics using non‐rigid substrates. In this context, nanomaterial‐modified conducting paper (CP) represents a new concept that utilizes paper as a functional part in various devices. Paper has drawn significant interest among the research community because it is ubiquitous, cheap, and environmentally friendly. This review provides information on the basic characteristics of paper and its functionalization with nanomaterials, methodology for device fabrication, and their various applications. It also highlights some of the exciting applications of CP in point‐of‐care diagnostics for biomedical applications. Furthermore, recent challenges and opportunities in paper‐based devices are summarized.

## Graphical abstract

**The basic characteristics of paper and its functionalization with nanomaterials, as well as methodology for device fabrication and their various applications** are presented. It also highlights some of the exciting applications of conducting paper in point‐of‐care diagnostics for biomedical applications. Furthermore, recent challenges and opportunities in the development of paper‐based devices are summarized.

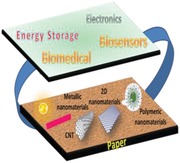

## 1 Introduction

Point‐of‐care (POC) devices are being widely explored for many clinical and analytical applications. POC devices are useful for speedy decision on additional testing or therapy as compared to conventional laboratory assay methods. Since the testing can be performed at resourceless area and/or near the patient, therefore POC has attracted much attention for health monitoring.^1^ Currently, the majority of the POC devices are based on glass, ceramics, or semi‐conducting substrates that are neither flexible nor biocompatible and are thus not helpful for the fabrication of advanced POC devices. Recently, research is being focused on the development of flexible sensors that have applications in healthcare. These POC devices are likely to be cost‐effective, portable, flexible, and user‐friendly with distinct advantages of safe disposal and biosafety management.[qv: 1c,2] Moreover, these devices can be interfaced with curved surfaces, complex geometries, soft and non‐planar body surfaces.

Flexible devices are an interesting alternative to the bulky health‐monitoring devices due to their inexpensiveness, excellent flexibility, and lower manufacturing cost. In recent years, many review articles have discussed the characteristics of the flexible devices such as functional textiles, wearable devices,^3^ electronic skins,^4^ flexible bio‐devices and optoelectronics.^5^ In the field of biosensors, many handy and wearable non‐flexible biosensors have been reported, that are used for the measurement of biophysical parameters (heart rate, blood pressure, temperature, pH), and air quality.^6^ Research has also been focused on the use of flexible devices for biosensor applications wherein the biological elements are used for the sensing of biological and chemical entities. In this context, CP can play an interesting role in the ongoing development toward the development of flexible, lightweight, portable, cost‐effective, and disposable POC devices. There is also a possibility to enhance the properties of CP‐based devices by modifying it with functional nanostructured materials that are known to exhibit unique physiochemical properties.^7^ Nanomaterial modified CP can be utilized to improve the conduction of charges (electronic and ionic) within the 3D ordered structure of cellulose paper.^8^ Several inorganic and organic nanomaterials have been reported for the fabrication of various CP‐based devices.^9^

The performance of paper‐based device depends on the various properties such as surface roughness, mechanical strength, ink absorbance, and composition.^10^ Paper has a high surface‐to‐volume ratio that allows storage of increased amount of reagents. Their mesoporous structures provide faster transport of electrolytes and can be used to the ion diffusion in energy storage devices. Thus, paper with strong adsorption of desired electrolyte, favorable porous structure, and good stability in electrochemical and mechanical performance are preferred for the various energy storage applications. However, for electronics application high surface roughness is a negative factor that adds to increased printing difficulty and can affect the electrical conductivity. A smooth surface and less/non‐absorbent paper like photo‐paper, parchment, and wax paper have been found suitable for electronics application. Such surfaces are made by an additional coating of pigment (containing sodium polyacrylate, carboxymethyl cellulose, and starch), treatment of paper pulp, and wax coating, respectively, followed by calendaring.^11^ These steps make paper surface smooth and reduce the pore size. The strong absorbance, surface functionalization, biocompatiblity, and white background are some of the deciding factors for the various biomedical applications. In scaffolds and cell culturing experiment, biocompatibility is a major concern. However for measurement of physiological signal (ECG, pulse rate, etc.), strong and uniform attachment of interesting materials are important factors because they provide high surface area and low electrical impedance.^12^ For biosensor application, it is recommended that paper should have properties to transport fluid flow, high surface to volume ratio, efficient conductivity, and mechanical strength during electrochemical measurements. The most commonly used paper is filter/chromatography paper #1 that consists of 98% of α‐cellulose, uniformity on both sides, medium flow rate, and 0.18 mm thick.[qv: 10a] Further surface modification/treatment helps to attach desired biomolecules covalently on paper surface for enhanced biosensor performance. However, the aforementioned properties are dependent on a variety of environmental factors such as humidity, temperature, composition, etc. Therefore, a careful consideration is required prior to the selection of a suitable substrate. The paper has been mostly explored in microfludic‐based devices for fluid handling and analysis. In these devices, it is important to direct the fluid flow within the confined channel. Various hydrophobic materials such as wax, photoresist, paraffin, etc., have been used to make a hydrophobic barrier. Later it was integrated with various detection methods to obtain analytical information. Microfludic‐based paper devices are a separate study and various review articles are available in literature.^13^

This review explores the recent developments in nanomaterials modified CP for POC diagnostics. The significant characteristics and problems in the design and modification of flexible devices are discussed to highlight the features that affect the performance of CP‐based devices. Besides this, overview of different types of materials and fabrication techniques used for the development of CP are presented. Efforts will also be made to summarize some of the other applications of conducting paper including energy storage, printable electronics.

### 1.1 Conducting Paper (CP)

Paper is a flexible, lightweight, low cost, recyclable, and biodegradable material. The cellulose fibers present in a paper have diameter of 10–50 µm and length up to a few millimeters (2–5 mm). The intra‐intermolecular hydrogen bond leads to the formation of the microfibrillar structure that provides excellent mechanical properties to cellulose fibers. Cell walls of cellulose fibers consist of smaller fibrils. These smaller fibrils comprise of microfibril, hemicellulose, and lignin.[qv: 10c,11,14] The microfibrils are crystalline in nature and are mostly composed of cellulose ((C_6_H_10_O_5_)*_n_*) and cellulose molecules that consist of a linear chain of D‐glucose units that are linked together by β(1→4) glycosidic bond. However, lignin (a hydrophobic and more crossed linked polymer) and hemicelluloses (a hydrophilic polymer of shorter chain of different sugar molecules) are amorphous in nature and provide rigidity, brittleness, and yellow color over time. The proportion of lignin, hemicellulose, and cellulose vary across different types of paper.

Paper was initially used for packaging, displaying, and storing information. However, in due course of time, mankind has discovered applications in filtration (Whatman paper), transformer (as a dielectric material), and has been used as an actuator. Recently filter paper has been utilized as a substrate for the development of low‐cost, flexible sensor^11^ since it is known to have sufficient roughness, poor chemical and mechanical barrier that enable it to absorb conducting materials into its porous structure. Moreover, the paper substrate can be chemically modified to incorporate a wide variety of favorable functional groups that can modulate the bulk and surface properties of paper.^2^, ^15^

Since ancient times paper has been used for a number of analytical applications. The traditional paper tests are based on visual observation that mostly gives qualitative or semi‐quantitative results. The change in color of paper is an indication of positive or negative result.^2^, ^16^ The first commercial paper test for blood glucose detection called Dextrostix was developed by Ames Private Ltd. followed by the launch of pregnancy test kit by Unipath.^2^, ^17^ A US patent was filed on laminated assay device for detection of cholesterol by modifying paper substrate with hydrophobic printing.^18^ Recently, paper has been re‐introduced as a promising substrate for fabrication of flexible, disposable, lightweight, cost‐effective devices. Thanks to the advancement in technique and unusual properties of nanomaterial that may made it possible.^19^

Researchers have recently conceptualized a new dimension of paper‐based electronics that can reduce the cost of device fabrication. Apart from this, its flexibility, light weight, and ease of availability and disposability may fulfill the increasing demand for smart electronic devices. CP has been found to be a promising substrate for transport of electronic as well as ionic charge carriers. CP has received enormous attention for various technological applications, i.e., energy storage (capacitors and batteries), electronics (displays, thin film transistors, and touch pads) to biomedical devices (ELISA, cell culture, and biosensors).^11^, ^20^

### 1.2 Conducting Materials and Fabrication Methods

**Figure 1:**
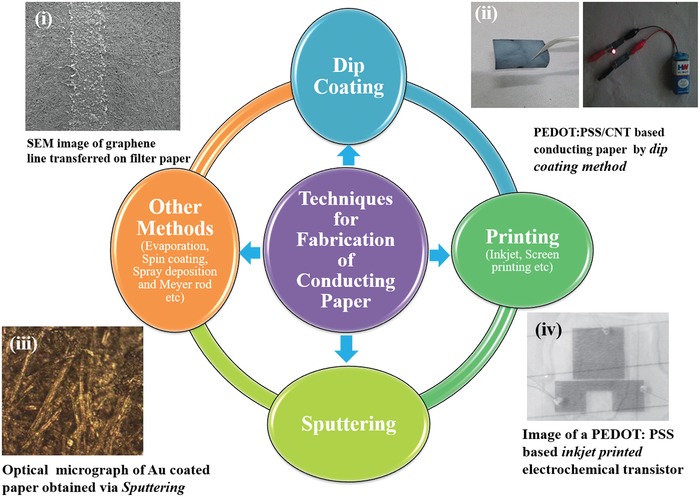
Fabrication of CP via different technique. Reproduced with permission.^23^ a) Copyright 2016, AIP Publishing. b) Copyright 2017, IOP Publishing. c) Copyright 2013, Wiley‐VCH. d) Copyright 2015, RSC Publishing.

Many materials including both organic and inorganic have been used to fabricate CP. The inorganic materials (metal, metal oxide, etc.) provide better electrical signal but are costly, and difficult to process. Besides this, cracks may result in these films during bending/sintering. On the other hand organic materials (conducting polymer, carbon nanomaterials, etc.) provide excellent flexibility, low cost, and solution processability but suffer from poor conductivity and/or long‐term stability. To overcome these limitations, a compromise can perhaps be made by using a composite material. Siegel et al. used a variety of metals (Al, Zn, Cu, Pb, Ni, Sb, Sn, Ti, Ag, Bi, In, Au, and Pt) to produce electrical conductive pathways on paper and studied its electrical conductivity, mechanical properties, melting point, and cost, etc.^21^ Besides this, organic materials can be utilized to make paper conducting.^22^ A variety of techniques (depending on the material behavior) have been used to deposit conducting materials over the paper. These include dip coating, printing, sputtering, spin‐coating, etc. (**Figure** **1**). Most of these processes require conducting materials or their templates.

**Figure 2:**
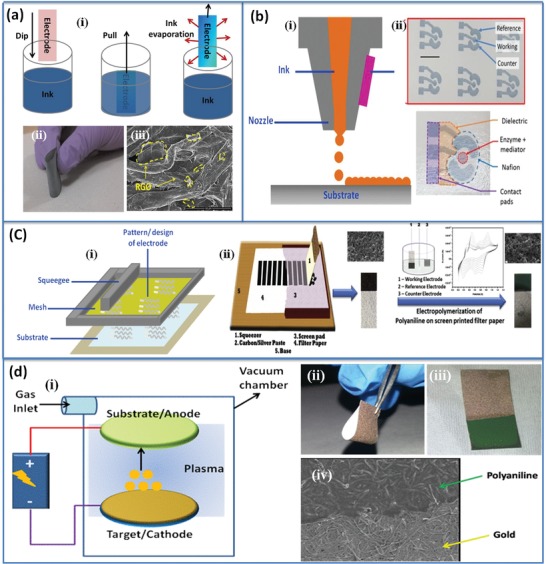
a) i) Schematic of dip‐coating method. ii) An image of PEDOT:PSS and RGO‐modified paper. This CP was fabricated by dip‐coating. iii) SEM of PEDOT:PSS/RGO‐coated filter paper. Reproduced with permission.^27^ Copyright 2015, Elsevier. b) i) Schematic of inkjet printing method. ii) Image of a fully‐inkjet‐printed glucose sensor on paper. The device has been fabricated by printing of PEDOT:PSS, dielectric layer and biological components. Reproduced with permission.^24^ Copyright 2018, Springer Nature. c) i) Schematic representation of screen‐printing method. ii) A screen‐printed CP electrode was fabricated using graphite and silver ink. Further, polyaniline was deposited on CP strips via electrochemical method to enhance electrochemical properties and anchoring of biomolecules. Reproduced with permission.^25^ Copyright 2012, Elsevier. d) i) Schematic of sputtering technique. Target materials were deposited (from the cathode) on the substrate placed over the anode in a vacuum chamber filled with usually inert gases. The thickness of the deposited layer could be controlled by the duration and intensity of discharge. ii) Image of gold‐sputtered filter paper. iii) Next, polyaniline was electrochemically deposited on gold‐coated paper to prevent cracks in the film during bending. iv) SEM image of polyaniline‐modified CP. Reproduced with permission.^26^ Copyright 2016, AIP Publishing.

Dip coating technique has often been employed to fabricate a CP using conducting ink or sol–gel precursors. In this method, the substrate is immersed and withdrawn into a reservoir of liquid with precise control (**Figure** **2**a). The thickness and reproduciblity of the film can be controlled by tuning the ink properties, functionality of the substrate, submersion time, dipping cycle, and humidity. Many materials such as carbon nanotubes, gold nanowires, PEDOT:PSS, and organic–inorganic nanostructured materials (PEDOT:PSS‐RGO, PEDOT:PSS‐CNT, PEDOT:PSS‐Fe_2_O_3_, etc.) have been employed to fabricate CP. Figure 2a (ii and iii) shows the optical and SEM image of a PEDOT:PSS‐RGO nanocomposite–coated paper.

Printing technique widely used for fabrication of flexible electronic components is considered to be rapid and low‐cost. Selection of paper substrate is essential to print suitable ink that can be easily adsorbed onto paper, and form uniform continuous layer. However, formulation of ink is a crucial step to print electronic component on a paper substrate. Properties of ink depend primarily on ink concentration, solvent used, and the presence of binders or additives, which define the viscosity, surface tension, and evaporation rate of ink. Usually binders or additives reduce the electrical performance of ink and choice of solvent is also limited in printing technique. The printing technique basically depends on the properties of ink and characteristics of substrate (surface energy, roughness, and porosity). The most common techniques are inkjet printing and screen printing. The advantage of using paper as a substrate is the fast drying of the ink, better adhesion of desired materials, homogenous printing, and large print thickness. Furthermore, low cost and environment friendly materials and solvent should be used in manufacturing process to obtain a recyclable and environment friendly product. Since the cellulose fibers are hygroscopic in nature and hence these may cause swelling of the substrate during the printing process that may affect the resolution of printing and adhesion of subsequent ink layers. Recently, a fully inkjet printed paper–based electrochemical device was fabricated for glucose detection (Figure 2b).^24^ All the components (electrodes, dielectric layer, biological coating etc.) were fabricated by inkjet method. In the screen printing method, inks were squeezed through a patterned screen mesh using squeezer. The patterned screen mesh contains small size of pore through which conducting ink was patterned onto the substrate (Figure 2c). The thickness of ink deposited on the substrate and print resolution depends on ink properties and the density of pores on patterned mesh. Mostly silver, graphite, and their composites were used to render electrical conductivity to the paper. However, it is possible to further modify the surface of a CP strip. A polyaniline film was recently deposited onto CP strips via electrochemical method (Figure 2c, ii) to enhance electrochemical properties and anchoring of biomolecules.[qv: 1c,25]

Paper can be made conducting by directly depositing a uniform layer of metal via sputtering technique. The main advantage of sputter deposition is that it can be easily used for even those materials that have high melting points. The target material may be a metal, alloy, ceramic, or compound. In case of the metal target, atoms can be ejected by the ionized gas for deposition onto any surface within the coating unit including the specimen, paper (Figure 2d, i). By adjusting and tuning the vacuum and the time of sputtering, thick coatings of metal depositions can be achieved on a paper substrate. Siegel et al. fabricated paper‐based printed circuit boards (PCBs) by sputtering silver and gold metals on the surface of a paper.^21^ Similarly, gold was sputtered on filter paper and electrochemically coated with polyaniline to prevent cracks in the film during bending (Figure 2c, ii–iv).^26^ Further, this surface was used to anchor protein and for the detection of cancer biomarker, CEA.

To introduce electrical conductivity in the paper, other methods like evaporation,^28^ spin coating,^28^ spray deposition,^29^ and the Meyer rod^30^–based coating have been reported. Compared to sputtering technique, the thermal evaporation method requires expensive equipment and high vacuum condition. For example, deposition of Au and Ag can be done via thermal evaporation while spray deposition can be used to deposit Ni and Ag. Spray deposition is a fast and cheap method for deposition of a metal. This method does not require any vacuum condition and deposition can be performed at room temperature and can be utilized for testing a device/design before considering the expensive technique such as evaporation and sputtering. As compared to evaporation and sputtering, spray coating has been found to have poor resolution and lower conductivity. A Meyer rod–based method has also been used to coat CNT and Ag nanowire ink on commercial Xerox paper.^30^ The Meyer‐rod is made of a stainless steel and a groove of certain diameter is present on it. This helps in conformal coating of the slurry‐based materials on a paper substrate. This technique is cheap, user‐friendly, and maintenance‐free. It is, however, difficult to get small size feature and the resolution is poor.

## 2 Application of Nanomaterial‐Modified CP

**Figure 3:**
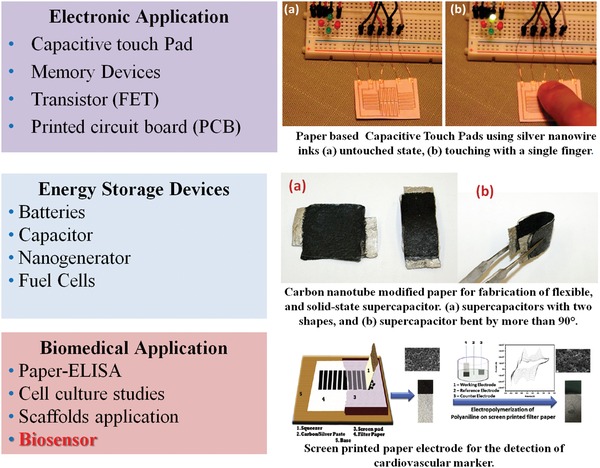
Application of CP in various fields. Reproduced with permission.^25^, ^33^ Copyright 2012, Elsevier. Copyright 2014, American Chemical Society. Copyright 2012, AIP Publishing.

The research and development of nanomaterial modified paper has dramatically intensified due to potential applications in electronics,^31^ energy storage devices,[qv: 31a,32] and biomedical and biosensors devices (**Figure** **3**). Integration of nanomaterials with paper is predicted to exhibit unique physical and chemical properties (i.e., thermal, electrical conductivity, optical properties, high mechanical strength, and electrochemical behavior) that may perhaps be due to the presence of a nanomaterial, flexibility, lightweight, low‐cost, disposability, and environment friendliness of paper. In the next section, we discuss the different potential applications of nanomaterial‐modified paper including electronics, energy, and medical diagnostics.

### 2.1 Electronic Applications

The recent developments in preparation of functional nanomaterials in the form of nanocrystals, nanowires, and nanotubes have led to the development of functional inks. These can be easily integrated with paper substrate and form a conductive layer. The CP can be used as a flexible platform onto which an electronic structure can be fabricated. For example, Lyth et al. reported homogenously coated paper substrate with multiwalled CNT for photodiode application.^34^ Härting et al. demonstrated the use of a traditional screen printing approach for the fabrication of paper‐based field effect transistor using interconnecting silicon nanoparticles.^35^ Manekkathodi et al. deposited a thin film of ZnO onto paper substrate for the fabrication of the flexible diode and UV photodetectors.^36^ Silver nanowire integrated paper was used to fabricate paper‐based capacitive touch pads[qv: 33a] and polyimide‐based printed circuit board (PCB) was replaced by paper‐based flexible PCB by using silver and gold metals on the surface of a paper.^21^ These unusual combinations of novel unique nanomaterials and paper indicate that a wide variety of electronic devices can be fabricated. Morphology and composition of the paper substrate have also been found to play a key role to construct high efficient devices. Lee et al. reported a highly conductive paper via low temperature (110 °C) stamping process using aluminum precursor ink [AlH_3_{O(C_4_H_9_)_2_}].^37^ The observed conductivity was found to be as good as thermally evaporated aluminum film and displayed excellent flexibility. Further, aluminum ink was deposited on various paper substrates and showed different electrical and mechanical performance. This was attributed to the different morphology and composition of the paper substrate.

The efforts are being made to replace glass based thin film transistor (TFT) screens with a less expensive, lightweight, flexible, and thermally stable substrate. Fortunato et al. developed an oxide‐based semiconductor thin film FET transistor wherein paper was used as a dielectric layer. Hybrid FETs with device on both sides of the paper were developed with excellent characteristics like high channel saturation mobility of >30 cm^2^ Vs^−1^ and subthreshold swing of 0.8 V dec^−1^.^38^ With recent advancements in flexible electronics, printed paper–based memory devices (PPMDs) have gained much attention. Lien et al. at National Taiwan University (NTU) worked on PPMDs and successfully fabricated a printed paper–based resistive random access memory (RRAM) device on A4 sheet with storage capacity in gigabytes.^39^ The PPMD showed excellent endurance, reliable retention, and operating capability under extreme bending condition. Unlike regular silicon‐based storage devices, PPMDs showed excellent ease of data handling, disposability, switching endurance, and reliable retention. Gong et al. fabricated a highly sensitive pressure sensor based on gold nanowire integrated conductive tissue paper.^40^ Conductive paper was sandwiched between PDMS and interdigited electrode integrated PDMS chip for fabrication of wearable sensor that easily monitored the blood pulse and could be used to measure small vibration forces originate from music.

There is a growing interest in exploiting the potential of paper as a substrate for ultrahigh frequency and microwave applications since these are inexpensive, reliable, and has durable wireless radio frequency identification (RFID) tags. RFID tags were used in logistics, monitoring of supply chain, healthcare, pharmaceutical, space, and anti‐counterfeiting. Another work relating to RFID based on paper has been reported by Yang et al., integrated with silver nanoparticles.^41^ This silver‐modified paper‐based RFID tags successfully reduced its cost and is eco‐friendly. Transparent and conducting electrodes are major elements for various optoelectronics application such as solar cell, touch panel, electrochromics, etc. The conventional devices are based on rigid and brittle glass substrate that limits their application for fabrication of smart electronic devices. Kang et al. fabricated a conductive and transparent paper using nanocellulose and conducting materials, i.e., silver nanowire and carbon nanotube for fabrication of foldable electrochromics devices that showed strong endurance to deformation, good cycling stability, and improve coloration efficiency.^42^

The electrical conduction in a modified paper is due to the presence of charge carriers (ions and electrons), and their short range mobility. The charge carriers are believed to be heterogeneous, involving many species as the paper material is chemically heterogeneous.^43^ Additionally paper is chemically and mechanically stable in normal atmospheric conditions and its excellent ability to absorb ink makes it superior as compared to other substrates. Its bulk and surface properties can be easily tuned by modifying the fiber, adding chemical additives, and surface treatment.^44^ Furthermore, the abundance of cellulosic material on this planet makes conductive paper a renewable and sustainable component for various electronic devices mentioned above such as circuit board, capacitive touch pad, memory devices, field effect transistor, frequency identification tags (FID), and electrochromic. However, humidity and temperature have been found to influence the properties of CP. Compared to traditional electronic devices, life span of CP‐based devices is currently the major issue. Nevertheless, with advancement in manufacturing technique it should be possible to make paper fiber resistant to external factor up to a certain limit and improve the device characteristics for longer period.

### 2.2 Energy Storage Devices

The 3D hierarchical structure of cellulose paper has the ability to store and transport liquid electrolytes. Its interconnected pores allow fast movement of the ionic species. These properties were used as a key for development of paper‐based energy storage devices. Further conducting paths were integrated into a paper using conducting materials such as metal oxides, nanowires, carbon nanomaterials, and conducting polymers.^11^, ^45^ These nanomaterial‐based CP have been predicted to have applications in energy storage devices such as electrochemical batteries,^46^ lithium‐ion batteries,^47^ supercapacitors,^48^ biofuel cells,^49^ and nanogenerators.^28^ Lithium‐ion batteries and supercapacitors are known to be excellent power sources for high power electronics whereas nanogenerators are useful for wearable electronics. Biofuel cells have proved handy for small power electronics such as microfluidics paper analytical devices (µPADs) and biosensors wherein the energy requirement is minimal.

Compared to other substrates, paper has exceptional properties to absorb conducting ink and form a stable film. It also minimizes the additional steps required for coating process and significantly reduces the device cost. The light weight of CP can be used as an excellent current collector in lithium‐ion batteries to substitute the metallic chemical counterpart. Wang et al. developed a flexible single walled CNT/poly‐cellulose paper for lithium‐ion battery with first discharge capacity of 153.3 mA h g^−1^ at 0.1 C and discharge capacity of 102.6 mA h g^−1^ at 10 C.^47^ Chou et al. integrated MnO_2_ nanowires with CNT paper using cyclic voltammetry for demonstration of super‐capacitor application.^48^ The supercapacitor showed specific capacitance of 167.5 F g^−1^ at a current density of 77 mA g^−1^. The composite paper retained 88% of the initial capacitance even after 3000 cycles of charge and discharge. Furthermore, CNT and silver nanowire composites were integrated with paper to fabricate a high efficient supercapacitor and lithium‐ion battery.^30^ The efforts are being made to fabricate small power devices that can utilize mechanical energy to convert it into electrical energy. Nanogenerators have proved to be an ideal power source for self‐powered system as an energy source for application in micro‐electro‐mechanical systems (MEMS). Kim et al. fabricated the first paper‐based mechanical nanogenerator.^50^ In this work, a piezoelectric active layer of ZnO rods was introduced on cellulose substrate that produced a current density of 2 µA cm^−2^ and an output voltage of 75 mV with stable current output after 10 mechanical bending cycles. In another work Zhong et al. designed a paper‐based nanogenerator using polytetrafluoroethylene (PTFE)–Ag–paper assembly.^28^ By applying external mechanical force on PTFE–Ag–paper assembly (nanogenrator) a maximum instantaneous power of up to ≈90.6 µW cm^−2^ was generated, which is sufficient to glow 70 LEDs simultaneously. An environment friendly triboelectric nanogenerator comprising of cellulose nanofibers (CNF) and Ag nanowires (AgNW) were reported.^51^ By contacting and moving apart two sheets of fabricated paper (AgNW/CNF) produced a power output of 693 mW m^−2^ against a 10 MΩ external resistance. The fabricated electrode (AgNW/CNF paper) could be easily decomposed into its constituents by simple sonication method and is hence, a clean energy source for low power devices. Microbial fuel cell (MFC) devices are using organic matter to generate electrical power. Here bacteria decomposed into organic matter and resultant chemical energy was used to generate electricity. Typically, MFCs are made up of two chambers (anode and cathode) that are separated by a membrane. During bacterial decomposition of organic matter H^+^/positive ions release and migrate from anodic to cathode chamber and resulted into flow of electricity. Electrical power generated by MFC was very low (≈3–4 W m^−2^) to meet the societal need. Fraiwan et al. developed the first paper‐based microbial fuel cell (MFC) with rapid electricity generation, an improvement over the conventional MFCs with long startup time usually several days to a week.^49^ Choi and coworkers developed battery stack using MFC that was able to glow a red LED for 30 min.[qv: 20a] This paper battery is simple, cost‐effective, and is a user friendly power source. It could also a source of power for single time run of paper‐based diagnostic devices. There is enough scope for MFCs since even O_2_ could be used as electron acceptor instead of conventionally used toxic ferricyanide.

One of the major concerns in paper‐based energy storage devices is the fast drying of electrolytes. Thus, sealing of device would be an alternative. Further, to increase the life span of these devices we can choose ionic electrolyte that could perhaps be utilized for longer duration. However, the transport of ions is usually low.

### 2.3 Biomedical Applications

The fluidic properties, 3D geometry, white background, biocompatible surface, ability to separate analyte, low cost, and easy to dispose off paper make them an ideal platform for biomedical applications.^2^, ^52^ Integration of a nanomaterial with paper may lead to improved quality of the paper‐based devices such as enhanced separation, color contrast, and loading of the biomolecules, etc. The nanomaterial‐modified paper has been predicted to have applications in bioassays,^53^ drug screening,^54^ ELISA,^55^ cell culture studies,^56^ scaffolds,^57^ and biosensing^58^ applications.

**Figure 4:**
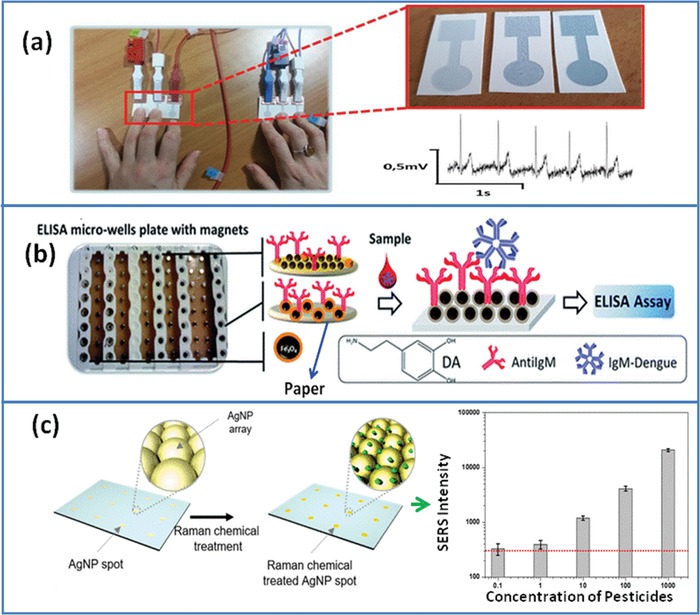
a) Electrophysiological signal measurement: A paper‐based electrocardiography was recorded using a PEDOT:PSS‐coated electrode via a simple finger‐to‐electrode contact. Reproduced with permission.^59^ Copyright 2017, Wiley‐VCH. b) ELISA: a polydopamine‐modified Fe_3_O_4_ nanoparticle was deposited on Whatman paper. The modified paper was used in pristine microwells having 96 well plates that are coupled to a magnet. Further, Fe_3_O_4_‐nanoparticle‐modified paper was used for the ELISA assay to detect dengue. The analytical performance of paper ELISA for IgM‐dengue detection shows two orders higher sensitivity (500 times lower detection limit) than traditional ELISA. Reproduced with permission.^60^ Copyright 2017, Royal Society of Chemistry. c) Analyte assay: a paper‐based SERS substrate was fabricated to detect pesticides (4‐ATP, thiram, and ferbam). The paper was initially treated with alkyl ketene dimer (AKD) to modify its property from hydrophilic to hydrophobic. This treatment increased the density of AgNPs and retention time on paper (SERS substrate) and therefore consequently enhances the sensitivity and reproducibility. Reproduced with permission.^62^ Copyright 2018, American Chemical Society.

For electrocardiogram (ECG) recording, the wet gel adhesives are needed that make them inconvenient for long‐term monitoring. Mostafalu et al. fabricated a patterned paper electrode using platinum, nickel, and copper nanowire. This nanomaterial‐modified flexible electrode can be utilized for recording of the ECG signal.^12^ The paper‐based electrodes provide high surface area and low impedance and can perhaps be helpful to record the signal even in dry attachment with skin. Similarly, Bihar et al. used PEDOT:PSS‐coated paper electrode to measure ECG signal via a simple finger to electrode contact (**Figure** **4**a) and electrode was stable for 3 months.^59^ This work has the potential to minimize the manufacturing steps and is cost effective. Besides this, it provides a metal free medical electrode with large durability. Kuzmenko et al. fabricated scaffolds by modifying electrospun cellulose with MWCNT and carbon nanofiber for enhanced neural tissue growth.^57^ It provides ideal feature similar to neural extracellular matrix that allows neural cell to grow, differentiate, and adhere to substrate. This kind of matrix is useful to regenerate neural tissue in vitro that will lead to increased understanding of the disease and will help to design new drug at laboratory scale. In another example, polydopamine‐modified Fe_3_O_4_ nanoparticles were deposited on Whatman paper termed as “Magnetic paper,” can be used for ELISA to detect dengue (Figure 4b).^60^ The analytical performance of paper ELISA for IgM‐dengue detection showed sensitivity (500 times lower detection limit) two order more than traditional ELISA method.

The surface modification of paper with nanomaterials reveals plasmonic behavior. Liu et al. fabricated a plasmonic filter paper by dipping paper in gold nanorods (GNRs) solution.^61^ This platform was used to differentiate normal and cancerous cells using surface‐enhanced Raman scattering (SERS) in which a diagnostic result based on the ratio of the spectral values was adopted to distinguish healthy and cancerous cells. Another work with paper‐based SERS substrate was employed for pesticides detection (Figure 4c).^62^ First, paper was chemically treated with alkyl ketene dimer to modify it from hydrophilic to hydrophobic. This treatment increased the density of AgNPs and retention time on paper and consequently enhanced the sensitivity and reproducibility. The electrocatlytic activity and functional group of nanomaterials were also taken as advantage for detection of various bioanalyte. Ornatska et al. modified the paper with ceria nanoparticles for naked‐eye detection of glucose.^63^ In this work, glucose oxidase was immobilized on the ceria nanoparticle‐modified paper. In the presence of glucose, the hydrogen peroxide genrated during enzymatic reaction and ceria modified paper induced a visual color change from white‐yellowish to dark orange. The change in the color intensity was directly proportional to glucose concentration. This assay utilized the redox behavior of cerium oxide as a colorimetric probe indicator and could be used for 10 consecutive cycles and stability of 79 d. In another work, zirconia nanotubes modified filter paper was utilized to bind oligonucleotides.^15^ The co‐ordination bond was formed between zirconia and the phosphate group present in probe DNAs. This platform (zirconia/paper) is highly sensitive and selective to detect complementary target DNA present at nanomolar concentration. Additionally, nanostructure ZnO‐coated paper possessed antibacterial activity.^64^ Besides this, CP can be used to culture other cells ranging from bacteria, fungi, plant to other human cells line such as primary, tumor, stem, osteoblasts, fibroblasts, and immune cells.[qv: 56b] These paper‐based platforms are compatible with standard analytical procedure that are usually used to monitor cell response in stress condition.[qv: 56b] Thus, paper can be an interesting and easy substitute for existing assay platforms since this would open up new possibilities in the biomedical field. However, strong and uniform attachment of material on paper substrate is very crucial because it provides high surface‐to‐volume ratio and allows low impedance. During measurement of physiological signal, compatibility of materials with human skin is very important to prevent unwanted allergic reaction.

### 2.4 Biosensor Applications

The CP‐based biosensors are recently gaining much interest for development of flexible, portable, low cost, power efficient, and environment friendly devices. These devices are known to have potential application in diagnostics, monitoring of analytes, and environmental studies. Wang et al. used single‐walled CNT to modify filter paper for environmental toxin detection (microcystin‐LR).^65^ Carvalhal et al. sputtered gold on Whatman paper for fabrication of electrodes and further, this paper electrode was used for quantitative detection of electroactive compounds, such as ascorbic acid and uric acid.^66^ Later these researchers used electrochemical method for detection of paracetamol and 4‐aminophenol using patterned gold electrode in the paper‐based microfluidic device.^67^ In another work, a screen printed paper electrode was fabricated using graphite and silver ink on the paper substrate followed by electrochemical deposition of polyaniline for the detection of cardiac (cTnI) and cancer biomarkers (sIL2Rα).[qv: 1c,25] The conventional laboratory assay requires long time and expertise; whereas these devices are fast, low cost, and easy to handle. Additionally, it does not require any large infrastructure and hence testing can be performed near the vicinity of patient.^1^ For the fabrication of an electrochemical biosensor, conducting electrodes such as glassy carbon, indium tin oxide, and gold‐coated glass electrodes can be used.^68^ These electrodes are rigid, brittle, and are costly which limit their applications for fabrication of smart, flexible, low cost, and wearable POC devices. In addition to these problems, conventional electrodes require high temperature for processing and expertise. On the other hand, CP‐based biosensors have attracted significant interest due to their light weight, flexibility, portability, high sensitivity, fast response time, and disposibility.[qv: 20b,27,53] CP has many advantages such as cost, renewable material and has strong adsorption properties that can be useful for the modification of paper for efficient fabrication of the biosensing devices.^2^ It has been considered to be an efficient platform that can conduct both electrons and ionic charge carriers. These two properties play a major role in electrochemical biosensor for communication with biomolecule during sensing process.[qv: 20b] The advantages and scope of CP as a sensor substrate are listed in **Table** **1** and are compared with conventional electrodes.

**Table 1:** Comparison between conventional electrode and CP electrode

S.No.	Properties	Conventional electrodes (Indium tin oxide, gold coated glass, Glassy carbon)	CP
1	Cost	High	Low
2	Flexibility	No	Yes (Room for improvement)
3	Transparency	Only ITO	Room for development
4	Biocompatibility	No	Room for development
5	Modification/Functionalization	Difficult	Easy
6	Fluid flow	Forced	Capillary action
7	Surface by volume ratio	Low	High
8	Fabrication and high throughput production	No	Yes
9	Disposability	Complex	Simple

**Figure 5:**
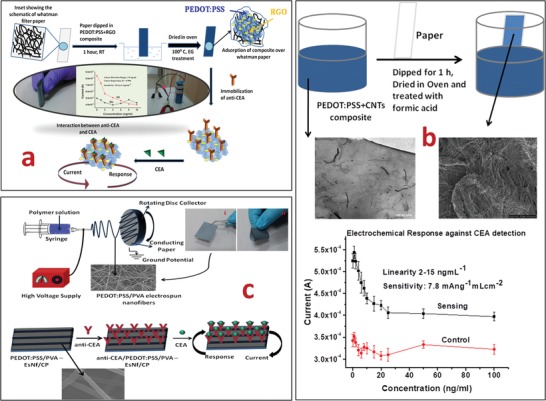
a) A graphic representation of PEDOT:PSS‐RGO‐modified CP. The fabricated CP shows excellent flexibility, electrochemical properties, and is used for CEA detection. Reproduced with permission.^27^ Copyright 2015, Elsevier. b) The schematic representation of PEDOT:PSS‐CNT paper by dip‐coating method. The PEDOT:PSS‐CNT paper electrode is further used for CEA detection. Reproduce with permission.^23^ Copyright 2015, Royal Society of Chemistry. c) CP modified by electrospinning of PEDOT:PSS/PVA‐nanofibers. Reproduced with permission.^71^ Copyright 2016, Wiley‐VCH.

Immobilization of bioreceptor on transducer surface is an important parameter for fabrication of efficient biosensing platform. For this purpose, an immobilization matrix can be used since it integrates and maintains the functionality of bioreceptor at one end and at the other end it binds with the transducer surface. It provides the accessibility of analyte to bioreceptor and, thereafter, physiochemical changes are measured by transducer. In this context, the incorporation of a nanomaterial as an immobilization matrix improves the characteristics of the CP in terms of performance, signal stability, biomolecules loading, and sensitivity.^69^ The large surface area of the nanomaterial‐integrated paper is likely to provide a better matrix for the immobilization of desired biomolecules leading to increased loading. The effect of immobilization matrix on the sensing performance of CP has been shown and discussed in **Figure** **5**. The solution processed CP comprising PEDOT:PSS‐RGO (Figure 5a) and PEDOT:PSS‐CNT (Figure 5b) was used to detect cancer biomarker, CEA in the range of 2–8 ng mL^−1^ with sensitivity of 25.8 mA ng^−1^ mL cm^−2^ and 2–15 ng mL^−1^ with sensitivity of 7.8 mA ng^−1^mL cm^−2^, respectively.[qv: 23a,27] These changes in sensing parameter relied on selection of the dopant (nanomaterials) and solvent. Conductivity of CP was found to be increased by two orders of magnitude (10^−4^ to 10^−2^ Scm^−1^) by treatment with different solvents like ethylene glycol (EG), formic acid, and DMSO.[qv: 23a,27,70] Whereas nanomaterials such as RGO, CNT, and *n*Fe_2_O_3_ can be utilized to obtain improved electrochemical properties, signal stability, and enhanced mass loading of a bioreceptor. In another example, morphology of the CP was modified by electrospinning of PEDOT:PSS/PVA‐nanofibers.^71^ The outcome of the electrochemical response studies showed that the PEDO:PSS/PVA‐EsNf modified CP could be used to estimate CEA in the range of 0.2 to 25 ng mL^−1^, with sensitivity of 14.2 µA ng^−1^ mL cm^−2^ and shelf life of 22 d (Figure 5c). The resulting paper sensor covered the entire physiological range of CEA secreted in serum sample (<3 to 20 ng mL^−1^) with acceptable lower detection limit. These nanomaterial‐modified CP electrodes exhibited improved results as compared to conventional ITO, gold, and glassy carbon electrode. Next, these CP platforms could be easily decomposed by simple burning/incineration that are an additional advantage for biomedical waste management. **Table** **2** summarizes the materials and techniques used for the fabrication of nanomaterials modified paper‐based electrochemical biosensors and their characteristics as reported in the literature. In spite of these interesting developments, there is a considerable scope for the development of simple, low cost, flexible, lightweight, and environment‐friendly biosensors with improved sensing characteristics using nanomaterial‐modified paper platform.

**Table 2:** CP‐based electrochemical biosensors: fabrication and characteristics

S.No.	Substrate	Materials	Fabrication method	Analyte	Detection technique	Sensing parameter	Refs.
1	Whatman filter paper #1	Screen printed carbon electrode	Drop casting	Glucose	Amperometry	[L] = 1–5 × 10^−3^ m [LOD] = 0.18 × 10^−3^ m [T] = 4 months	72
2	Whatman filter paper #1	Ag/AgCl ink, carbon ink, wax, graphene, AuNP	Wax printing, screen printing	DNA	Differential pulse voltammetry	[L] = 0.0008–500 × 10^−12^ m [LOD] = 0.2 × 10^−15^ m [T] = 4 months	73
3	Whatman filter paper #1	Ag/AgCl ink, carbon ink, SU‐8	Photolithography, screen printing	Glucose Pb(II)	Chronoamperometry Anodic striping voltammetery	Glucose [L] = 0–22.2 × 10^−3^ m [LOD] = 0.22 × 10^−3^ m [S] = 0.43 µA mM^−1^ mm^−2^ Pb(II) [L] = 5–100 ppb [LOD] = 1 ppb [S] = 0.17 µA ppb^−1^	74
4	Whatman filter paper #1	Graphene, polyvinyl pyrrolidone, polyaniline (G/PVP/PANI), carbon ink, Ag/AgCl ink	Electrospraying wax printing screen printing	Cholesterol	Amperometric	[L] = 0.05–10 × 10^−3^ m [LOD] = 1 × 10^−6^ m [S] = 34.77 µA mM^−1^ cm^−2^ [T] = 2 weeks	75
5	Platinum sputtered glass	Nitrocellulose paper, polyester cellulose blend	Drop casting	Glucose	Amperometric	–	76
6	Whatman paper #1	ITO coated glass wax, Prussian blue	Drop casting	Glucose	Electrochromic	–	77
7	Filter paper	Gold nanorods	Dip coating	Oral squamous cell carcinoma (OSCC) cell line CAL‐27	Surface‐enhanced Raman scattering (SERS)	Intensity ratio of particular Raman peak	61
8	Filter paper	Carbon nanotube (CNT)	Dip coating	Microcystin‐LR (MC‐LR)	Amperometry	[L] = 1–10 ng mL^−1^ [LOD] = 0.6 ng mL^−1^	65
9	Whatman filter paper #1	Screen printed carbon electrode	Drop casting	Pb(III)	Anodic striping voltametry	[L] = 10–100 ppb [T] = 3 months	78
10	Whatman filter paper #1	Gold	Sputtering	Uric Acid (UA) Ascorbic Acid (AA)	Amperometry	[L] = 0–40 pmols [LOD] = 0.02 mmol L^−1^ AA [S] = 152 nA L mmol^−1^ UA [S] = 64 nA L mmol^−1^	66
11	Whatman filter paper #1	Carbon ink	Screen printing	Glucose lactate uric acid	Chronoamperometry	Glucose [L] = 0–100 × 10^−3^ m [LOD] = 0.21 ± 0.02 × 10^−3^ m [S] = 64 µA mM^−1^ Lactate [L] = 0–50 × 10^−3^ m [LOD] = 0.36 ± 0.03 × 10^−3^ m [S] = 40 µA mM^−1^ Uric acid [L] = 0–35 × 10^−3^ m [LOD] = 1.38 ± 0.13 × 10^−3^ m [S] = 6 µA mM^−1^	79
12	Whatman filter paper #1	Carbon ink, prussian blue	Wax screen printing	Glucose	Chronoamperometry	[L] = 0.5–5 × 10^−3^ m [S] = 1 µA mM^−1^	80
13	Whatman filter paper #1	Carbon ink	Solid wax printing screen printing	Glucose	Chronoamperometry	[L] = 0–20 × 10^−3^ m [LOD] = 0.35 × 10^− 3^ m [S] = 0.041 µA mM^−1^	81
14	Whatman filter paper #1	Gold and platinum NPs on SPE	Wax printing	Single stranded DNA	Amperometry cyclic voltammetry	[L] = 10.0 × 10^−15^ m–100 × 10^−9^ m [LOD] = 6.3 × 10^−15^ m	82
15	Whatman filter paper #1	Pencil‐drawn electrode, carbon paste	Wax printing	Ascorbic acid, dopamine, paracetamol	Amperometry	–	83
16	Whatman filter paper #1	Graphite pencil	Wax printing	Glucose	Chronoamperometry	[L] = 0.01–1.5 × 10^−3^ m [LOD] = 0.38 × 10^−6^ m [T] = 5 d	84
17	Whatman filter paper #1	CdS quantum dot and CNTs on SPE	Wax printing	Adenosine triphosphate (ATP)	Cyclic voltammetry	[L] = 1–1000 × 10^−12^ m [LOD] = 0.2 × 10^−12^ m [S] = 27 nA pM^−1^ [T] = 4 weeks	85
18	Whatman filter paper #1	Gold NPs on carbon	Wax printing, SlipPAD technique	Single stranded DNA, Thrombin	Alternating current voltammetry	ssDNA [LOD] = 30 × 10^−9^ m Thrombin [LOD] = 16 × 10^−9^ m [T] = 4 weeks	86
19	Japanese paper	Screen‐printed electrode	Screen printing	Glucose	Amperometry	[L] = 10–100 × 10^−3^ m [S] = 0.055 µA mM^−1^	87
20	Whatman filter paper #1	Gold NPs on SPE	Wax printing screen printing	Microcystin‐LR	Differential pulse voltammetry	[L] = 0.01–200 µg mL^−1^ [LOD] = 0.004 µg mL^−1^ [T] = 2 months	88
21	Whatman filter paper #1	Carbon graphite ink on SPE	Screen printing	Nicotinamide adenine dinucleotide (NADH) Nitrite	Cyclic voltammetry	NADH [L] = 10–100 × 10^−3^ m [LOD] = 1.8 × 10^−6^ m Nitrite [L] = 10–100 × 10^−3^ m [LOD] = 15.1 × 10^−3^ m	89
22	Whatman filter paper #1	Chitosan–silver on SPE	Screen printing	Cancer antigen 125 (CA125) Carcinoma antigen (CA199)	Square wave voltammetry	CA125 [L] = 0.1–100 U mL^−1^ [LOD] = 0.02 mU mL^−1^ [S] = 2.56 µA mL U^−1^ [T] = 21 d CA 199 [L] = 0.1–100 U mL [LOD] = 0.04 mU mL^−1^ [S] = 0.91 µA mL U^−1^ [T] = 21 d	[qv: 33a]
23	Whatman filter paper #1	Gold–manganese oxide NPs on SPE	Wax printing screen printing	Prostate protein antigen (PSA)	Differential pulse voltammetry	[L] = 0.005–100 ng mL^−1^ [LOD] = 0.0012 ng mL^−1^ [T] = 4 weeks	90
24	Whatman filter paper #1	Polyaniline (PANI) on SPE, graphite and silver paste	Screen printing	Human troponin I	Cyclic voltammetry	[L] = 1–100 ng mL^−1^ [S] = 5.5 µA ngmL^−1^ cm^−2^	25
25	Whatman filter paper #1	SPE, graphite, carbon, and silver paste	Screen printing	2‐(dibutylamino) ethanol NADH	Cyclic voltammetry	DBAE [L] = 3–5000 × 10^−6^ m [LOD] = 0.9 × 10^−6^ m NADH [L] = 0.2–10 × 10^−3^ m [LOD] = 72 × 10^−6^ m	91
26	Whatman filter paper #1	Nafion/graphene oxide on SPE	Photolithography screen printing	DNA mismatches	Electro chemiluminescence	[L] = 10 × 10^−9^ m–5 × 10^−6^ m [LOD] = 1 × 10^−9^ m [T] = 3 months	92
27	Whatman filter paper #1	Tin oxide QDs/ RGO/gold NPs on SPE	Screen printing	ATP	Electrochemical impedence spectroscopy [EIS]	[L] = 0.1 × 10^−12^ m 100 × 10^−9^ m [LOD] = 0.025 × 10^−12^ m [T] = 5 weeks	93
28	Filter paper	SWCNTs	CNT ink painting	Human immunoglobulinG (HIgG)	Amperometry	[L] = 6.3–62 × 10^−12^ m [S] = −70.8 pA pmol^−1^ sL^−1^	94
29	Whatman filter paper #1	PEDOT:PSS‐RGO	Dip coating	CEA	Chronoamperometry	[L] = 2–8 ng mL^−1^ [S] = 25.8 µA ng^−1^ mL cm^−2^ [T] = 21 d	27
30	Whatman filter paper #1	PEDOT:PSS‐CNT	Dip coating	CEA	Chronoamperometry	[L] = 2–15 ng mL^−1^ [S] = 7.8 µA ng^−1^ mL cm^−2^ [T] = 18 d	[qv: 23a]
31	Whatman filter paper #1	PEDOT:PSS‐nFe_2_O_3_	Dip coating	CEA	Chronoamperometry	[L] = 4–25 ng mL^−1^ [S] = 10.2 µA ng^−1^ mL cm^−2^ [T] = 34 d	70
32	Whatman filter paper #1	Gold, PANI	Sputtering, electrochemical coating	CEA	Chronoamperometry	[L] = 2–20 ng mL^−1^ [S] = 13.9 µA ng^−1^ mL cm^−2^ [T] = 22 d	26
33	Whatman filter paper #1	PEDOT:PSS/PVA electrospun nanofiber	Dip coating	CEA	Chronoamperometry	[L] = 0.2–25 ng mL^−1^ [S] = 14.2 µA ng^−1^ mL cm^−2^ [T] = 22 d	71
34	Office paper (Fabriano, Italy, 80 and 100 gm/m^2^)	Carbon black‐ Prussian blue nanocomposites (CB/PBNPs), Ag/AgCl ink, graphite ink, wax	Wax printing, screen printing	Ethanol	Amperometry	[L] = 0–10 × 10^−3^ m [LOD] = 0.52 × 10^−3^ m [S] = 9.13 µA mM^−1^ cm^2^	95
35	Filter paper and nitrocellulose membrane	Ag/AgCl ink, carbon ink, wax, CB/PBNPs	Wax printing, screen printing	Nerve stimulant (Paraxon)	Amperometry	[L] = 5–25 µg L^−1^ [LOD] = 3 µg L^−1^ [T] = 15 d	96
36	Whatman filter paper #1	AuNP‐modified Cu‐based MOFs, Au‐coated paper, carbon ink, Ag/AgCl ink, wax	Wax printing, screen printing	miRNA‐155	DPV	[L] = 1 × 10^−15^ m–10 × 10^−9^ m [LOD] = 0.35 × 10^−15^ m	97
37	Filter paper (Cordenons, Italy, 67 g m^−2^)	Prussian blue nanoparticles (PBNPs), carbon ink, Ag/AgCl ink, wax	Wax printing, screen printing	Glucose	Amperometry	[L] = 0–30 × 10^−3^ m	98
38	Screen printed electrode (SPE)	Silane functionalized silica nanoparticles/ banana peel tissue (source of enzyme)/ mediator modified filter paper disc	Drop casting	L‐tyrosine	DPV	[L] = 0.05–600 × 10^−6^ m [LOD] = 0.02 × 10^−6^ m	99
39	Whatman filter paper #1	Cyclodextrin functionalized AuNPs (CD@AuNPs), carbon ink, Ag/AgCl ink, wax	Wax printing, screen printing	CEA and PSA	DPV	CEA [L] = 0.005–100 ng mL^−1^ [LOD] = 0.002 ng mL^−1^ PSA [L] = 0.002–100 ng mL^−1^ [LOD] = 0.001 ng mL^−1^	100

Abbreviations: [L] = linear detection range, [LOD] = lower detection limit; [S] = sensitivity; [T] = stability; Ag/AgCl = silver/silver chloride; SPE = screen‐printed carbon electrode; ITO = indium tin oxide; NP = nanoparticle; CdS = cadmium sulfide; CNT = carbon nanotubes; SWCNT = single‐walled carbon nanotubes; PAD = paper analytical device; QD = quantum dot; RGO = reduced graphene oxides; DNA = deoxyribose nucleic acid, PANI = polyaniline.

CP‐based biosensor provides an interesting platform for simple, precise, and quick recognition of desired biomolecules. These CP‐based devices can be used as cost‐effective technology for the development of POC diagnostic systems that can be used in developing countries, where there is no availability of fully equipped facilities and trained human resources. The important benefit of using CP as a biosensing platform is their compatibility with chemicals and biochemicals.^101^ Nowadays efforts are being made toward the fabrication of paper‐based microfluidics devices that can be readily available and may play a crucial role in biomedical research.^102^ Prior to commercialization of CP‐based biosensors, many parameters such as accuracy and sensitivity must be addressed. And addressing other issues such as nonspecific adsorption of biomolecules, reduction in surface roughness, mechanical and environmental stability are very important for commercialization of these biodevices. Moreover, the application of CP‐based biosensors must not be limited to the detection and diagnosis of disease, the POC devices can also be used for the monitoring of toxins, and food quality, in environmental samples and agricultural applications. With advancement in fabrication techniques, novel nanomaterials, and structural design, it is anticipated that in the near future more exciting and innovative research in CP‐based biosensors will be seen.

## 3 Conclusion and Outlook

The recent development in conducting paper‐based devices has demonstrated exciting results ranging from electronics, energy storage to various biomedical applications. These conducting paper‐based substrates are gaining more popularity than the conventional substrates in terms of their cost, flexibility, availability, ease of modification, disposability, and easy to use. Further, the desired properties of these paper‐based devices can perhaps be achieved by specific modification of the paper substrate with nanomaterials. In spite of all these advantages, many fundamental and applied challenges hinder the commercialization of paper‐based electronic devices. For example, stability and durability of conductive paper‐based devices are currently lesser than those of traditional devices. Smooth surface, strong mechanical strength and novel unique materials may open the window for development of paper‐based electronic devices that have a wide range of application. Sealing of energy storage devices may prevent fast drying of the electrolyte and effect of humidity and temperature to some extent. In this context, enhanced cooperation between scientific research community and industrial partner is essential to develop paper for long‐term electronics application. Furthermore, controlled conditions are necessary for the fabrication of CP‐based POC devices as the properties of paper gets altered with humidity and temperature. The porous structure and high absorbance of paper are beneficial for strong adhesion of nanomaterials and immobilization of bioreceptor. Besides this, paper may interfere with the electrical conductivity and non‐specific adsorption of other analytes that can yield false signal during measurement. These intrinsic surface properties could perhaps be controlled during manufacturing process and an application‐oriented paper may perhaps provide the desired solution. Besides this, in‐depth analysis of paper composition with geometric structure may open door to control the surface architecture. For qualitative or yes/no screening test, paper‐based devices are doing well. However, for quantitative detection poor sensitivity and lack of reproducibility are presently major concern. Thus, the development of more sensitive and accurate detection strategy would be very helpful to commercialize POC devices. The novel nanomaterials, advancement in fabrication technique and appropriate structural design may result in improved properties, accuracy, and sensitivity of the CP‐based POC devices.

## Conflict of Interest

The authors declare no conflict of interest.
